# Supplementary material for: A Global Screen for Assembly State Changes of the Mitotic Proteome by SEC-SWATH-MS
Source: Cell Syst. 2020 Feb 26;10(2):133–155.e6. doi: 10.1016/j.cels.2020.01.001 (PMC7042714; doi:10.1016/j.cels.2020.01.001)

O94992 | HEXI1_HUMAN | HEXIM1 CLP1 EDG1 HIS1 MAQ1
Monomer MW [kDa]: 40.623 Monomer expected elution fraction: 48

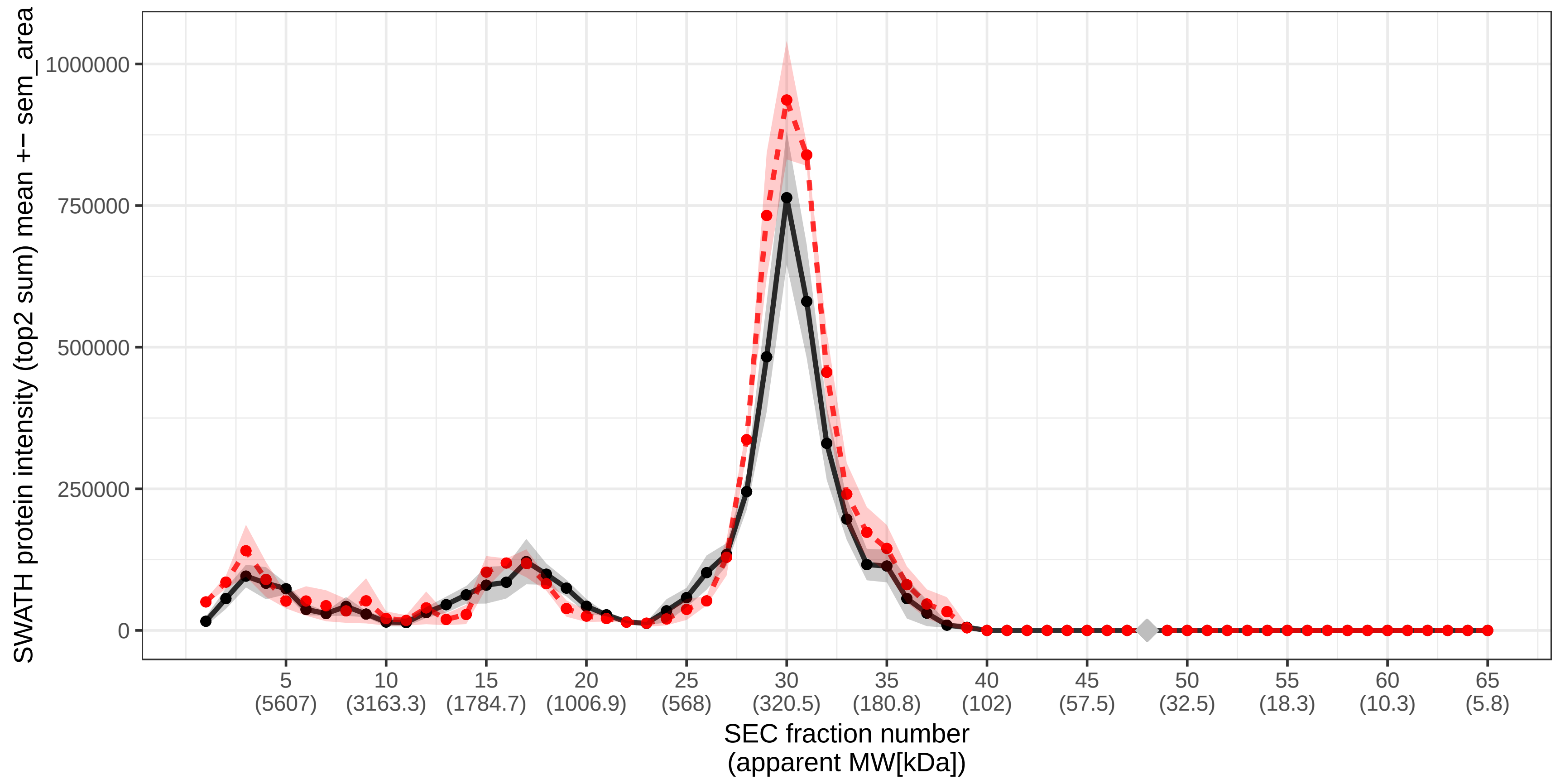

Supplement: Data S1. SEC-SWATH-MS Protein Chromatograms, Related to Figure 1 [file mmc6.zip › SECchrom_O94992_HEXI1_HUMAN_HEXIM1_CLP1_EDG1_HIS1_MAQ1.pdf]
